# Are peptidomimetics the compounds of choice for developing new modulators of the JAK-STAT pathway?

**DOI:** 10.3389/fimmu.2024.1406886

**Published:** 2024-06-24

**Authors:** Alessia Cugudda, Sara La Manna, Daniela Marasco

**Affiliations:** Department of Pharmacy, University of Naples “Federico II”, Naples, Italy

**Keywords:** jak-stat, peptidomimetics, macrocycles, inflammation, cytokines

## Abstract

Protein-protein interactions (PPIs) play critical roles in a wide range of biological processes including the dysregulation of cellular pathways leading to the loss of cell function, which in turn leads to diseases. The dysfunction of several signaling pathways is linked to the insurgence of pathological processes such as inflammation, cancer development and neurodegeneration. Thus, there is an urgent need for novel chemical modulators of dysregulated PPIs to drive progress in targeted therapies. Several PPIs have been targeted by bioactive compounds, and, often, to properly cover interacting protein regions and improve the biological activities of modulators, a particular focus concerns the employment of macrocycles as proteomimetics. Indeed, for their physicochemical properties, they occupy an intermediate space between small organic molecules and macromolecular proteins and are prominent in the drug discovery process. Peptide macrocycles can modulate fundamental biological mechanisms and here we will focus on peptidomimetics active on the Janus kinase/signal transducers and activators of transcription (JAK-STAT) pathways.

## 1 Introduction

The JAK-STAT pathway is pivotal in transmitting signals initiated by cytokines, including interleukins (ILs), during immune responses, inflammation, and cancer development (1, 2). Physiologically, cytokine stimulation leads to JAK-mediated phosphorylation of specific tyrosine residues on STAT proteins, located in SH2 domains. Phosphorylated STATs dimerize, translocate into the nucleus, and regulate gene expression, tuning inflammatory and immune-related genes (3, 4). The dysregulation of this pathway can occur through mutations in upstream oncogenes, cytokine receptors, JAK, or STAT proteins and are commonly associated with cancer progression: its selective upregulation in response to cytokines drives immune responses, inflammation, and carcinogenesis (5, 6). Hence, JAK-STAT is a key target for therapeutics (7) and understanding the endogenous regulatory mechanism of this pathway is essential for designing new drugs (8).

Small molecules inhibitors of JAK proteins (JAKi) have been FDA-approved as: tofacitinib (Pfizer) for nail psoriasis (9), ruxolitinib (Novartis) for primary myelofibrosis (PMF) (10), fedratinib (Celgene) for myelofibrosis (11), filgitinib (Galapagos) for rheumatoid arthritis (RA) (12), upadacitinib (AbbVie), baricitinib (Eli Lilly) and abrocitinib (Pfizer) for atopic dermatitis (AD) (13). Many studies concerning these drugs highlighted their off-target effects, particularly in patients defined as ‘at risk’ (i.e. ≥65 years), affected by cardiovascular problems, smokers or at high risk to develop cancer (14). To limit side effects, several attempts are made in the routes of administration and to potentiate their local effects. For example, JAKi for the treatment of asthma are inhaled (15) others for AD have been formulated to act topically, as delgocitinib (approved in Japan), which demonstrated more effective in the treatment of AD in pediatrics (16).

In addition, a regulatory feedback of this pathway includes the expression of Suppressor Of Cytokine Signaling (SOCS) proteins (17) which share a common mechanism of action (MOA) consisting in inhibiting JAK-STAT by competing with SH2 of STATs for the binding to JAKs, through their own SH2. In addition with this general MOA, SOCS1 and SOCS3 members contain a Kinase Inhibitor Region (KIR), which directly inhibits the kinase activity of JAKs acting as pseudo-substrate toward the kinase catalytic site (18). In addition these two proteins differently interact with JAK proteins: indeed from crystal structure studies while SOCS1 forms a binary complex with JAK1 (19), SOCS3 interacts simultaneously with JAK2 and the glycoprotein 130 (gp130) (20).

The downregulation of SOCS1 or SOCS3 has been observed in several diseases: SOCS1, for its role in interferons (IFNs) (21, 22) and interleukins (ILs) (23–25) signaling, in involved in inflammatory diseases as rheumatoid arthritis and psoriasis (26, 27) and atherosclerosis (28); while SOCS3 is involved in tumour development and its deficiency, relevant in triple-negative breast cancer (TNBC), is associated with a worse prognosis (29).

## 2 Peptidomimetics targeting JAK-STAT

Recently, resurgence has taken place in developing proteomimetics for therapeutic intervention: from the design of peptides mimicking the functions of proteins involved in diseases to the improvements of their drug-like features thanks to innovative synthetic and formulative platforms (30, 31). Proteomimetics, often in macrocyclic format, occupy an intermediate space between small organic molecules and macromolecules and combine significant binding affinities and selectivity, synthetic accessibility, low immunoreactivity and toxicities (32–34). Macrocyclizations render peptides more stable with increased membrane permeability, and stability in cellular environments (32, 35, 36).

Few but important examples of bioactive proteomimetics have been reported for several proteins of the JAK-STAT network paving the way to novel therapeutics (37).

**Figure 1:**
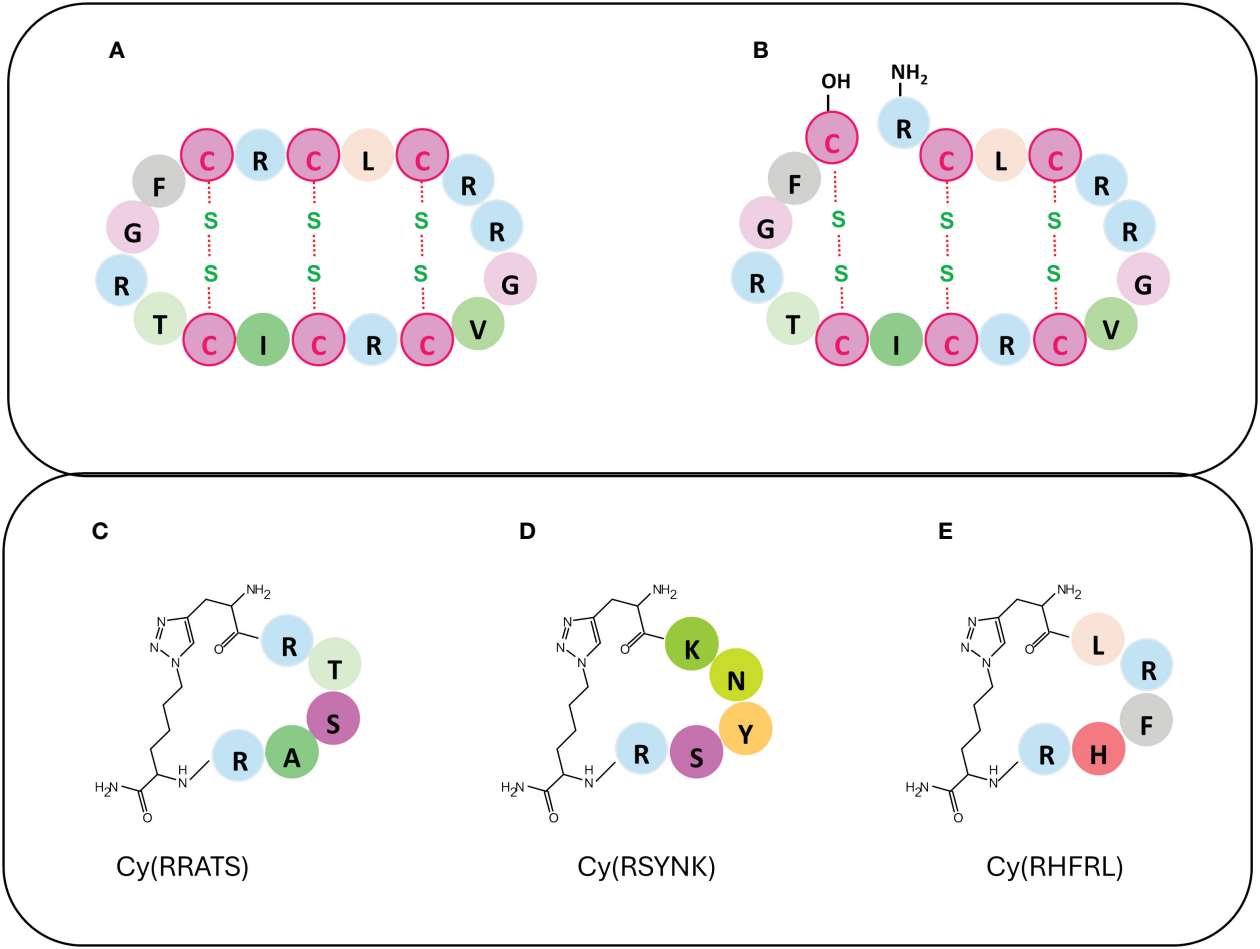
Upper panel: mimetics of θ-defensins **(A)** RTD-1, **(B)** acyclic RTD-1. Lower panel: mimetics of IL-17 SynEp **(C)** LF1, **(D)** LF2 mimicking IL17F and **(E)** LA3 mimicking IL17A.

IL-6 is a pro-inflammatory cytokine capable to activate several JAKs. Its dysregulation is associated with autoinflammatory and autoimmune diseases sepsis, irritable bowel syndrome (IBS), atherosclerosis, thrombosis, rheumatoid arthritis, and type 1 diabetes (38–40); hence the identification of inhibitors represents an interesting strategy to regulate its pathway (8). In this context, the θ-defensins are natural 18-mer macrocyclic peptides found in certain primates, such as rhesus macaques, able to regulate the production of cytokines, including IL-6, against various microbes (41, 42). Structurally, θ-defensins consist of a pair of antiparallel β-sheets linked by three disulfide bonds arranged as a ladder along the sheets to form an extremely stable structure (**Figure 1A**). The Rhesus θ-defensin-1 (RTD-1) suppresses the release of pro-inflammatory cytokines, as TNF-α and IL-6 (43). The RTD-1 isoform regulates the release of soluble tumor necrosis factor (sTNF) by inhibiting TNF-α-converting enzyme (TACE), which is a zinc metalloprotease responsible for cytokine production through proteolysis or “shedding” (43, 44). Aberrant TACE activity leads to increased TNF-α levels in inflammatory diseases and cancer progression (45–47). The macrocyclic structure of RTD is crucial for TACE inhibition, conversely, its structural modifications, as the absence of lactam bond head-to-tail RTD-1 (**Figure 1B**), cause the loss of inhibition (43). Hence one potential application could be the development of synthetic macrocyclic analogues of θ-defensin to block IL-6 production and limit TNF-dependent pathways in inflammatory diseases.

Within IL-17 family, there are six homodimers (A-F) and one heterodimer, A/F, known to interact with five receptors (RA-E) (48): in detail, IL-17A and F bind to the heterodimeric receptor complex formed by IL-17RA and RC (49). Different IL-17 proteins are linked to distinct biological activities i.e. IL-17A is associated with chronic obstructive pulmonary disease (COPD), while IL-17F in psoriasis and rheumatoid arthritis (50, 51).

To target these cytokines for therapeutic intervention selective drugs are required (52) and macrocyclic peptides mimicking IL-17A and F, have been reported. By using the *in situ* click screening method, unique epitopes were identified: Phe^40^-Ser^70^ for IL-17F and Ile^27^- Lys^61^ for IL-17A (53). From these, synthetic epitopes (SynEps) bearing clickable N-terminal tail were designed and analyzed and two of them, SynEp1 and SynEp2, resulted active against IL-17F while SynEp3 against IL-17A. Synthetic variants of SynEp compounds were generated through combinatorial approaches and screened obtaining macrocyclic binders highly specific (**Figures 1C–E**).

**Figure 2:**
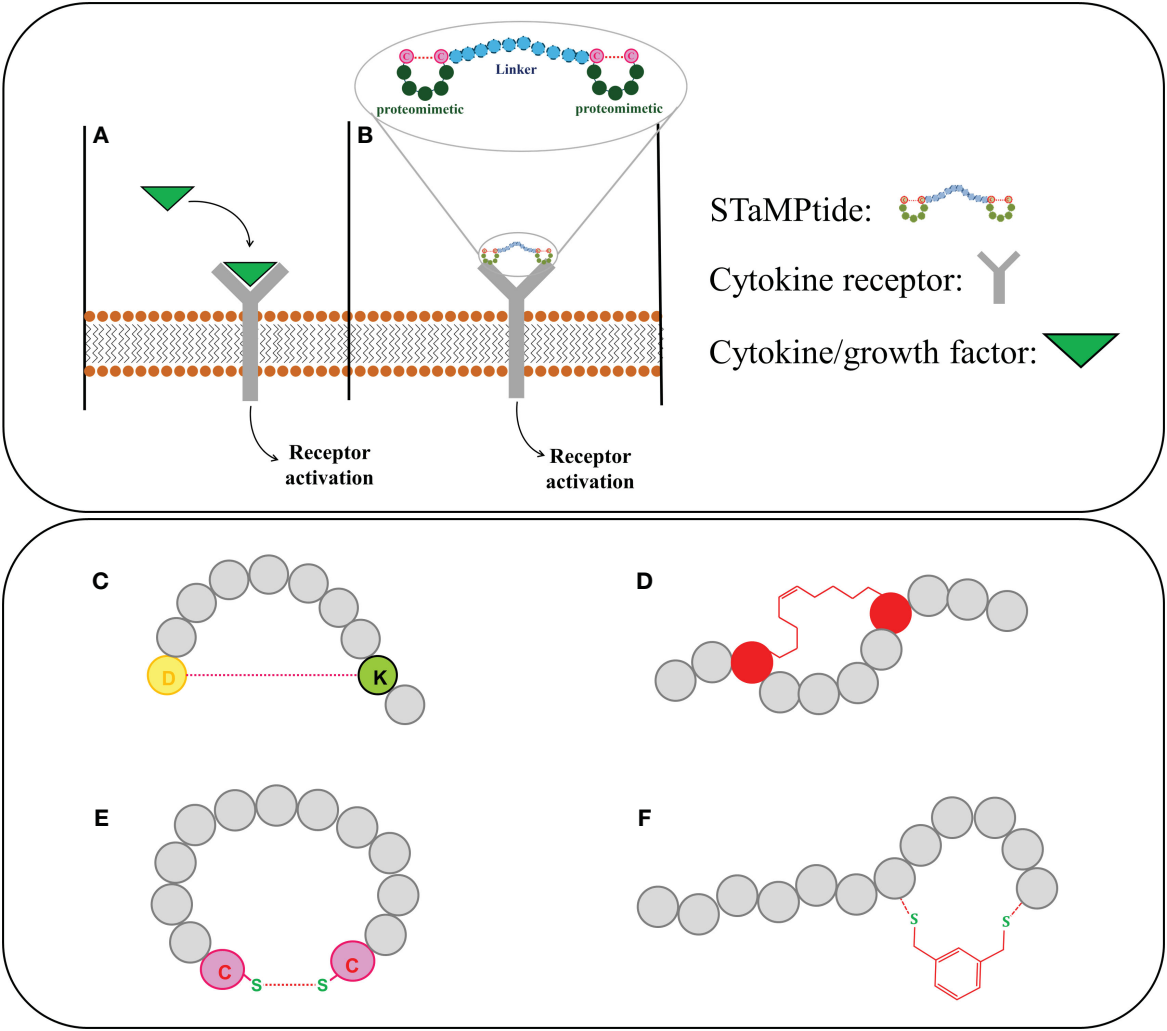
Upper panel: activation of cytokine receptors by **(A)** their endogenous ligands and **(B)** STaMPtide mimetics; Lower panel: different cyclization of peptide sequences: **(C)** amide, **(D)** hydrocarbon and **(E)** disulphide and **(F)** by introduction of non-native scaffolds.

Activators of JAK/STAT can be used as antiviral: type I interferons (IFN-α/β) are known to inhibit viral infection (54, 55), but interferons-based therapies have several side effects hence the need for new antiviral drugs (56). In patients with end-stage dilated cardiomyopathy (DCM), gp130 and JAK-STAT signaling are altered (57), studies conducted in left ventricular (LV) myocardia pointed out reduced levels of JAK2 phosphorylation and gp130 (57). The restoration of JAK/STAT in DCM is critical to prevent cardiomyocytes apoptosis and stimulates the expression of cardioprotective genes such as superoxide dismutase and vascular endothelial growth factor (58–61). Erythropoietin (Epo) and Thrombopoietin (Tpo) proteins activate JAK-STAT similarly to other cytokines through the binding to the corresponding receptors, EpoR and TpoR (**Figure 2A**) (62). In this way, Epo regulates bone marrow erythropoiesis and Tpo platelet production (63, 64) hence, the development of Epo and Tpo mimetic peptides (EMP and TMP) is valuable in diseases as erythrocyte and platelet disorders (65).

In general, an interesting approach to obtain cytokines mimetics is based on the design of Single-chain Tandem Macrocyclic Peptides (STaMPtides) which are constituted by two disulfide-cyclic peptides linked by peptide linkers (usually (Pro-Ala)_n_) (**Figure 2B**). Interestingly STaMPtides mimicking Epo and Tpo have been developed (66): two moieties of a mimetic of Epo, named EMP35 (67), were linked through 8- or 22-mer–Pro-Ala (PA) linkers, respectively. Both STaMPtides were able to activate the cascade EpoR/JAK2 and to induce the phosphorylation of JAK2 protein acting as strong mimetics of Epo (66). A similar approach was followed to design TMP-based STaMPtides: a mimetic of Tpo (68) was dimerized with an 8-mer PA linker to form TMP-PA8. Its activity was compared with that of recombinant human Tpo (rhTpo) using a phosphokinase array, in both cases the analysis of cell lysates revealed the activation of JAK2 protein (66).

An opposite therapeutic approach consists in engineering inhibitors of JAK-STAT pathway assuming as structural template natural endogenous regulators as SOCS proteins and hence the design and optimization of SOCS mimetics (69).

Concerning SOCS1, many studies demonstrated that the linear peptide covering the KIR domain inhibits/reduces: *i*) the activation of STAT by cytokines Th1 and 17 in leukocytes, *ii*) the activation and migration of vascular cells and macrophages *in vitro* (70), *iii*) the expression of cytokines with pro-inflammatory properties in atherosclerotic plaques (71), *iv*) the renal inflammation, oxidative stress and fibrosis (72), *v*) the chronic intraocular inflammatory disease (as uveitis) (73) (equine recurrent uveitis (ERU) (74, 75), *vi*) the abdominal aortic aneurysm (AAA) (76), and *vii*) the severity of skin lesions, autoantibody production and kidney disease in lupus-associated pathologies (77). In detail it demonstrated a protective role in glomerular changes in MsPGN rat models by reducing macrophage infiltration and inhibiting macrophage polarizing to the M1 phenotype (78). A SOCS1-KIR linear peptidomimetic, named PS5, was developed in our research group (79): in keratinocytes and explants of type-1 skin disorders demonstrated greater efficacy with respect to KIR (80)reduced the migration and proliferation (“wound healing”) of VSMCs with important antioxidant properties *in vitro* and *in vivo* (28). More recently, a lactame macrocyclization led to novel compounds named internal cyclic PS5 analogues (icPS5 and icPS5(Nal1), which bears the substitution Phe/Nal1, 1-Naphthyl-L-alanine) which inhibited JAK-mediated tyrosine phosphorylation of STAT1 and reduced cytokine-induced proinflammatory gene expression, oxidative stress generation and cell migration: in this context the Nal1 containing cycle exhibited long-time anti-migratory effects which are very important to limit plaque formation (81). More recently within icPS5 sequence, SAR investigations were carried out by performing crucial amino acids substitutions and/or modifications affecting the ring size: these studies confirmed the feasibility of this class of SOCS1 peptidomimetics, as specific inhibitors of JAK2 (82).

On the other hand, our research group was the first to develop SOCS3 peptidomimetics following a structure-based approach quite similar to that of SOCS1: a long peptide, called KIRESS, exhibited a good affinity for JAK2 and an efficient suppression of IL-22 signaling in keratinocytes, in athymic nude mice with squamous cell carcinoma (SCC) (83) as well as in primary tumour growth and pulmonary metastasis in triple negative breast cancer (TNBC) models (84). Similarly, into primary cultured cells, KIRESS reduced the Neural stem cells (NSCs) proliferation via blocking the cell cycle progression from the G0/G1 to S phase and attenuated astrocytic differentiation (85). In parallel, to explore different SOCS3 protein regions involved in JAK2 recognition, several chimeric peptides connecting non-contiguous protein regions, with a strongly aromatic fragment, were investigated: the derived mimetic, named KIRCONG chim, revealed able to recognize JAK2 exhibiting a low micromolar value of dissociation constant with good anti-inflammatory properties in VSMC and RAW264.7 macrophages (86). Its further development was limited by poor water solubility which has been recently overcame by the introduction of polyethylene glycol (PEG) moiety as spacer instead of the two β-Alanines of KIRCONG chim impressively suppressed NV (87).

With the aim to improve drug-like features of KIRCONG chim, we also investigated in the recent past (88) and currently (unpublished data) novel cyclic analogues bearing different chemical linkages among SOCS3 regions. In detail, head-to-tail macrocycles of KIRCONG chim endowed with, amide (**Figure 2C**), hydrocarbon (**Figure 2D**) and disulphide (**Figure 2E**) bonds demonstrated reduced affinity toward JAK2 and very limited water solubility (88). We are currently applying the so-called CLIPS (Chemical Linkage of Peptides on Scaffolds) strategy (89) in different local stretches of the KIRCONG chim sequence (**Figure 2F**). CLIPS is a versatile strategy and involves the cyclization of linear peptides via reaction of thiol-functionalities of the cysteines with a small rigid entity (unpublished data), but despite the easiness of cyclization, determines a reduction of water solubility with respect to its linear counterpart. Hence a fine tuning among affinity, stability and aqueous solubility should be taken into account in the development of novel SOCS mimetics.

## 3 Discussion

The proteomimetic approaches have predominantly focused on the folding features of protein interacting regions at PPIs interfaces, sometimes evolving toward unnatural structures with unprecedented features, as helical foldamers (90). More recently, proteomimetics have been developed for their use as biomimetic agents, selective binders or catalysts with promising applications in chemical, biological, medical, and material fields. In this context the number of biocompatible reactions used for the construction of proteomimetics is continuously in growth, as well as computational design algorithms (91). The cyclization of small molecules, peptides and macromolecules is a fundamental strategy to design precise 3D shapes tailored to chemical function and a major challenge, in current drug development efforts, is the generation of macrocycles targeting PPIs. To address this issue many, innovative design and synthetic strategies are in development including combinatorial, to diversify macrocyclic scaffolds (33) and screening formats (36). The JAK/STAT signaling pathway is characterized by extensive crosstalk of its components and is an important case study: it is endowed with many PPIs where an individual protein engages specific interactions (69). Both the activation and inhibition of JAK/STAT by external agents, in different pathological contexts, demonstrated therapeutic values. Herein we reported several examples to illustrate the importance of proteomimetic approach to selectively regulate this immune response signaling: in it mimetics of cytokines, acting at different pathway levels, amplify signaling cascades, leading to robust cellular responses in cell growth and differentiation regeneration and tissue repair. Conversely mimetics of negative regulators as SOCS1 and 3 are currently demonstrating growing therapeutic interest in inflammatory-related disorders and cancer, respectively.

## Data availability statement

The original contributions presented in the study are included in the article/supplementary material. Further inquiries can be directed to the corresponding author.

## Author contributions

AC: Conceptualization, Writing – review & editing. SL: Conceptualization, Writing – original draft. DM: Conceptualization, Writing – original draft, Funding acquisition, Writing – review & editing.
